# Cdc42 deficiency induces podocyte apoptosis by inhibiting the Nwasp/stress fibers/YAP pathway

**DOI:** 10.1038/cddis.2016.51

**Published:** 2016-03-17

**Authors:** Z Huang, L Zhang, Y Chen, H Zhang, Q Zhang, R Li, J Ma, Z Li, C Yu, Y Lai, T Lin, X Zhao, B Zhang, Z Ye, S Liu, W Wang, X Liang, R Liao, W Shi

**Affiliations:** 1 Southern Medical University, Guangzhou, Guangdong, China; 2 Department of Nephrology, Guangdong General Hospital, Guangdong Academy of Medical Sciences, Guangzhou, China

## Abstract

Podocyte apoptosis is a major mechanism that leads to proteinuria in many chronic kidney diseases. However, the concert mechanisms that cause podocyte apoptosis in these kidney diseases are not fully understood. The Rho family of small GTPases has been shown to be required in maintaining podocyte structure and function. Recent studies have indicated that podocyte-specific deletion of Cdc42 *in vivo*, but not of RhoA or Rac1, leads to congenital nephrotic syndrome and glomerulosclerosis. However, the underlying cellular events in podocyte controlled by Cdc42 remain unclear. Here, we assessed the cellular mechanisms by which Cdc42 regulates podocyte apoptosis. We found that the expression of Cdc42 and its activity were significantly decreased in high glucose-, lipopolysaccharide- or adriamycin-injured podocytes. Reduced Cdc42 expression *in vitro* and *in vivo* by small interfering RNA and selective Cdc42 inhibitor ML-141, respectively, caused podocyte apoptosis and proteinuria. Our results further demonstrated that insufficient Cdc42 or Nwasp, its downstream effector, could decrease the mRNA and protein expression of YAP, which had been regarded as an anti-apoptosis protein in podocyte. Moreover, our data indicated that the loss of stress fibers caused by Cdc42/Nwasp deficiency also decreased Yes-associated protein (YAP) mRNA and protein expression, and induced podocyte apoptosis. Podocyte apoptosis induced by Cdc42/Nwasp/stress fiber deficiency was significantly inhibited by overexpressing-active YAP. Thus, the Cdc42/Nwasp/stress fibers/YAP signal pathway may potentially play an important role in regulating podocyte apoptosis. Maintaining necessary Cdc42 would be one potent way to prevent proteinuria kidney diseases.

Podocytes are highly differentiated cells with complex actin cytoskeletal architecture that play a key role in maintaining the integrity of glomerular filtration barrier. Therefore, loss of podocyte will surely disturb the function of glomeruli. Apoptosis is one of the main reasons that causes loss of podocyte and sequentially induces proteinuria. Accumulating evidence show that podocyte apoptosis is one of the most important mechanisms in the pathogenesis of many kidney diseases, such as chronic kidney diseases,^1^ diabetic nephropathy,^2, 3^ focal segmental glomerular sclerosis,^4^ *et al.* Thus, preventing podocyte apoptosis will be a promising therapeutic target for treating these kidney diseases. However, the concert mechanisms that cause podocyte apoptosis in these kidney diseases are still far from being fully understood.

Rho family small GTPases RhoA, Rac1, and Cdc42 are the three most extensively studied prototypes, and are known powerful regulators of actin cytoskeletal dynamics, cell adhesive interactions, motility, or cell polarity.^5^ However, these Rho-GTPases also control other important cellular functions, such as gene expression, proliferation and apoptosis.^5, 6^ A large body of data implicates RhoA, Rac1, and Cdc42 in the pathogenesis of disease processes in the kidney including glomerular diseases.^6, 7, 8, 9, 10^ Recent studies also indicated that podocyte-specific deletion of Cdc42 *in vivo*, but not of RhoA or Rac1, leads to congenital nephrotic syndrome, podocyte foot process effacement and glomerulosclerosis.^11, 12^ However, whether Cdc42 is involved in podocyte apoptosis remains unknown. Meanwhile, further studies will also be required to unravel the detailed downstream signaling cascades of Cdc42 in podocytes and the exact mechanisms leading to podocyte apoptosis and proteinuria.

Yes-associated protein (YAP) is a major downstream cascade of the Hippo pathway which controls the expression of genes that promote cell proliferation and inhibits apoptosis.^13, 14^ Under normal condition, dephosphorylated YAP localizes in the nucleus and functions as a transcriptional co-activator that mainly through interacting with TEA domain family member family transcriptional factors to induce target gene expression.^15^ YAP phosphorylation promotes its cytoplasmic sequestration and inactivation.^16, 17^ Recent studies have indicated that YAP is an anti-apoptotic molecule in podocyte, and podocyte-specific deletion of YAP leads to proteinuria kidney diseases.^18, 19^ Moreover, loss of stress fiber caused by insufficient Cdc42 can result in reducing transcriptional activity and nuclear localization of YAP.^20, 21^ Therefore, it is likely that Cdc42 deficiency may induce podocyte apoptosis by reducing nuclear YAP.

In this study, we demonstrate that the loss of Cdc42 *in vitro* and *in vivo* by small interfering RNA (siRNA) and selective inhibitor ML-141, respectively, caused podocyte apoptosis and proteinuria, accompanied with increased pro-apoptotic Bax and decreased anti-apoptotic Bcl-2 gene and protein expression. Our results further demonstrated that insufficient Cdc42 or neuronal Wiskott–Aldrich syndrome protein (Nwasp), its downstream effector, could decrease the mRNA and protein expression of YAP, which had been regarded as an anti-apoptosis protein in podocyte. Moreover, our data indicated that the loss of stress fibers caused by Cdc42/Nwasp deficiency decreased YAP mRNA and protein expression, and induced podocyte apoptosis. Cell apoptosis induced by Cdc42/Nwasp/stress fiber deficiency was significantly inhibited in podocytes with overexpressed-active YAP. Therefore, the Cdc42/Nwasp/stress fibers/YAP signal pathway may potentially play an important role in regulating podocyte apoptosis. Maintaining necessary Cdc42 would be one potent way to prevent proteinuria kidney diseases.

## Results

### Apoptosis was significantly increased in high glucose (HG)-, lipopolysaccharide (LPS)- or adriamycin (ADR)-injured podocytes *in vitro* and *vivo*

**Figure 1:**
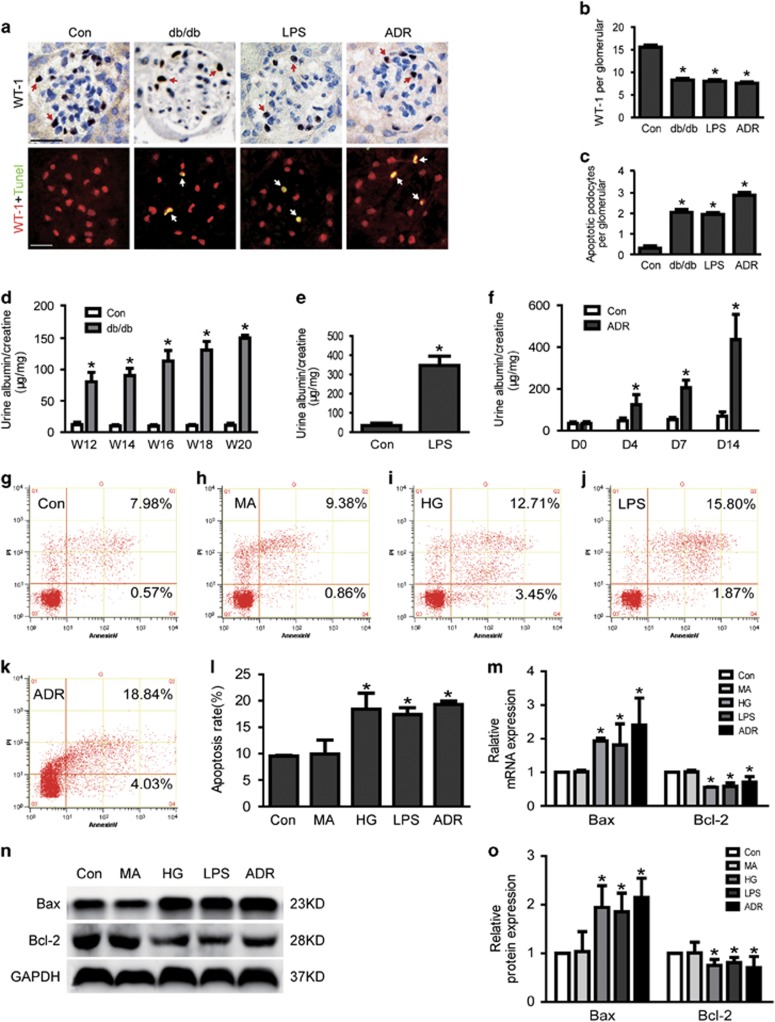
Apoptosis was increased in HG, LPS or ADR-injured podocytes *in vitro* and *in vivo*. (**a**) On top: typical images of immunochemistry stained (WT-1-positive cells, brown) glomeruli from control, db/db, LPS or ADR mice. Below: representative micrographs of dual-color fluorescence staining of kidney glomeruli for WT-1 (red) and TUNEL (green) from control, db/db, LPS or ADR mice. Magnification × 400, scale bar=25 *μ*m. (**b**) Quantification of podocyte markers WT-1-positive cells per glomerular section. The number of WT-1-stained nuclei was obtained from 20 glomeruli per kidney section from five mice per group. (**c**) Absolute count of the numbers of glomerular cells positive for both TUNEL and WT-1 (white arrows, apoptotic podocyte), data were expressed as the means of apoptotic podocytes from 20 randomly selected glomeruli. *P<0.05 *versus* Con, *n*=3. (**d–f**) Proteinuria was obviously increased in db/db, LPS or ADR mice compared to control mice. (**g–l**) Podocytes were stained with Annexin V/PI for flow cytometry analysis. Cell apoptosis rate was significantly increased in HG, LPS or ADR-treated podocytes comparing to normal controls. (**m**) Bax mRNA expression was increased as Bcl-2 was decreased in HG, LPS or ADR-treated podocytes. (**n–o**) Bax protein expression was increased as Bcl-2 was decreased in HG, LPS or ADR-treated podocytes. Podocyte apoptosis in MA (as an osmotic control) and the Con group show hardly any difference in all *in vitro* studies (**g–o**). Data were from at least three independent experiments. *P<0.05 *versus* controls

To confirm podocyte apoptosis in db/db mice or the mouse model of LPS or ADR-induced proteinuria and podocytopathy, podocyte apoptosis in glomeruli was examined by immunostaining with anti-WT-1 antibody, a podocyte-specific marker, and TUNEL. As shown in Figures 1a (on top) and b, there was a marked reduction in glomerular WT-1-positive cells in glomeruli of db/db mice, LPS mice or ADR mice relative to control mice. TUNEL staining (Figures 1a (below) and c) demonstrated that podocyte apoptosis was obviously increased in db/db mice, LPS mice or ADR mice compared to control mice (0.30±0.11 *versus* 2.05±0.14, 1.95±0.09 and 2.85±0.13, respectively, *n*=3, *P*<0.05). Consistent with the results above, urinary albumin excretion (*μ*g/mg creatinine), the parameters of proteinuria and podocytopathy, were also remarkably increased in db/db mice, LPS mice or ADR mice as compared with that in control mice (Figures 1d–f).

In addition, cell apoptosis was also further evaluated *in vitro*-cultured podocytes treated with HG, LPS or ADR. As shown in Figures 1g–l, cell apoptosis rates were 9.53±0.15, 9.93±2.64, 18.43±3.02, 16.59±1.97, 18.89±1.00% in control, mannitol (MA), HG, LPS and the ADR group, respectively. Consistent with the results above, the mRNA and protein expression of Bax, a well-recognized indicator of apoptosis were significantly increased in HG, LPS and ADR-treated podocytes comparing to normal controls (Figures 1m–o). However, Bcl-2, the indicator of anti-apoptosis was significantly decreased in HG, LPS and ADR-treated podocytes (Figures 1m and o). These data indicated that cell apoptosis was significantly increased in HG, LPS or ADR-injured podocytes *in vitro* and *in vivo*.

### Cdc42 expression and activity were decreased in injured podocytes *in vitro* and *in vivo*

**Figure 2:**
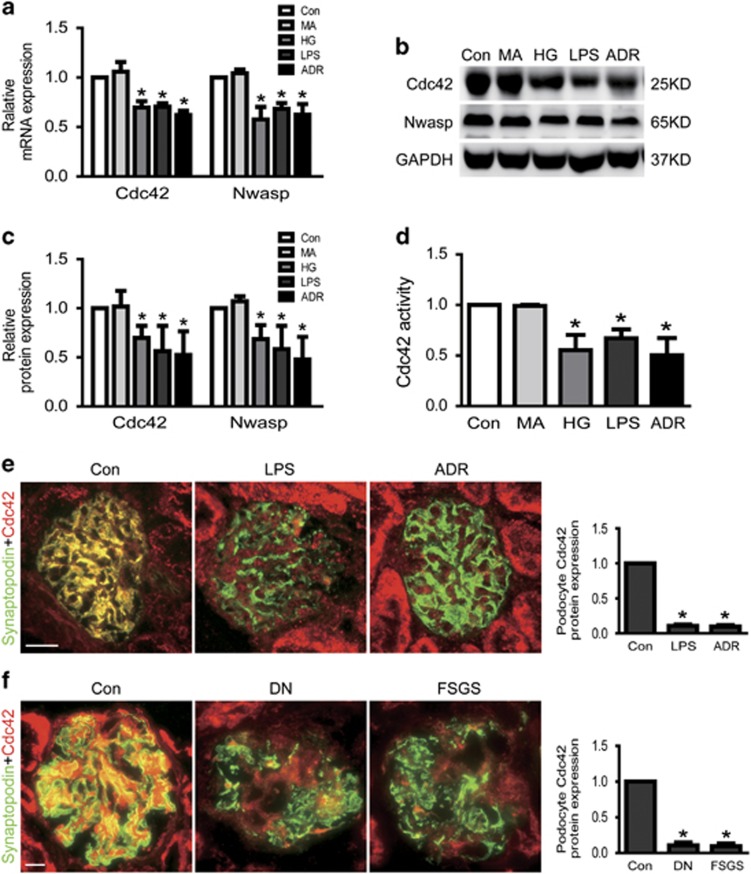
Cdc42 and Nwasp were decreased in injured podocytes. (**a**) The mRNA expression of Cdc42 and Nwasp were decreased in HG, LPS or ADR-injured podocytes. (**b, c**) The protein expression of Cdc42 and Nwasp were decreased in HG, LPS or ADR-injured podocytes. (**d**) Cdc42 activity was decreased in HG, LPS or ADR-injured podocytes. (**e**) Representative micrographs of dual-color fluorescence staining of kidney glomeruli for Cdc42 (red) and synaptopodin (green) from control, LPS and ADR mice. Magnification × 400, scale bar=25 *μ*m. (**f**) Representative micrographs of dual-color fluorescence staining of kidney glomeruli for Cdc42 (red) and synaptopodin (green) from control, DN and FSGS patients. Magnification × 400, scale bar=25 *μ*m. All *in vitro* studies result in MA (as an osmotic control) and the Con group show hardly any difference (**a–d**). All data were from at least three independent experiments. *P<0.05 *versus* controls

Cdc42 deletion results in massive neonatal proteinuria, kidney failure, and associated death within 2 weeks of birth, podocyte-specific RhoA and Rac1 deletion mutants are conspicuously healthy. Based on this, we explored that the Cdc42 expression and activity in injured podocyte *in vitro* and *in vivo*. As shown in Figures 2a–c, the mRNA and total protein expression of Cdc42 were significantly decreased in injured podocyte induced by HG (30 mM) or LPS or ADR comparing to normal controls. Similarly, Cdc42 activity was also obviously decreased in HG or LPS or ADR-treated podocytes. The percentages of Cdc42 activity were 100, 98.85±1.19, 55.52±15.29, 67.11±9.19, 50.34±16.97% in control, MA, HG, LPS and the ADR group, respectively (Figure 2d).

Next, we investigated Cdc42 expression in LPS or ADR-treated mice, which the former was well-recognized proteinuric mouse model,^22, 23^ the latter was a well-known experimental model of human proteinuria disease resemble to FSGS.^4, 24^ Immunofluorescent staining exhibited Cdc42 expression was decreased in glomerular podocyte of LPS or ADR-injected mice, comparing with vehicle control mice, respectively (Figure 2e). Using immunofluorescent staining, we also investigated the expression of Cdc42 in glomerular podocytes of patients with nephropathy (DN) or focal segmental glomerulosclerosis (FSGS). Consistent with the results from mice, in patients with DN or FSGS, the protein expression of Cdc42 was also markedly decreased in glomerular podocytes (Figure 2f).

### Loss of Cdc42 caused podocyte apoptosis and proteinuria

**Figure 3:**
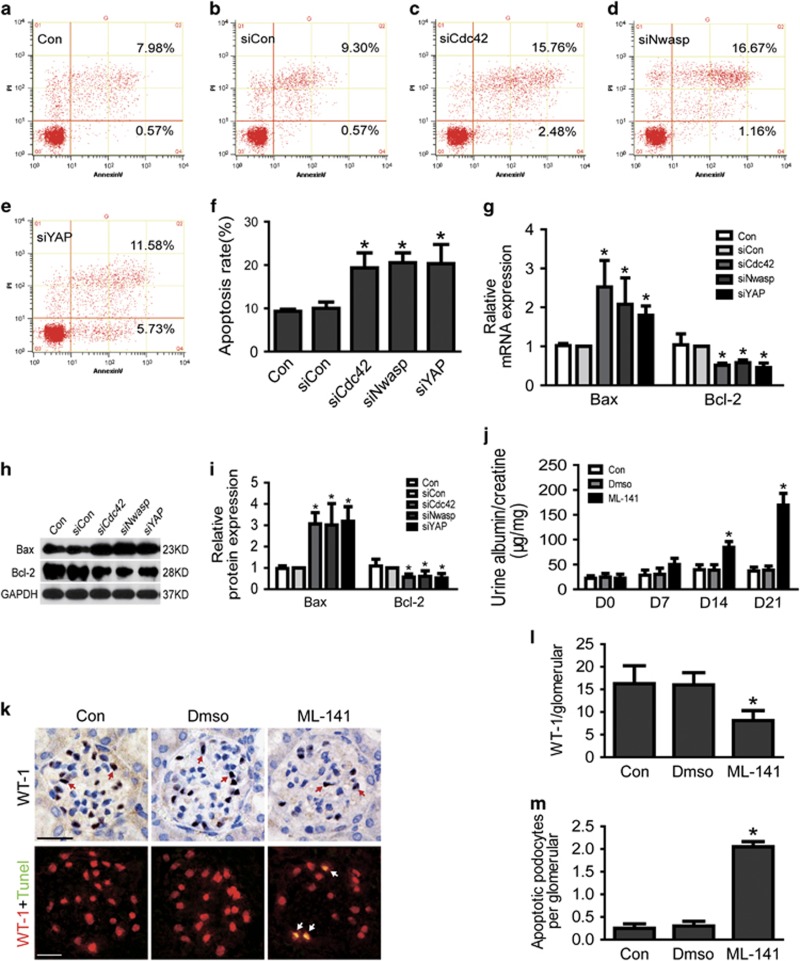
Loss of Cdc42, Nwasp or YAP-induced podocyte apoptosis. (**a–f**) Podocytes were stained with Annexin V/PI for flow cytometry analysis. Cell apoptosis rate was significantly increased in Cdc42, Nwasp or YAP knockdown podocytes. Con, siCon, siCdc42, siNwasp, siYAP were short for blank control, control siRNA, Cdc42 siRNA, Nwasp siRNA, YAP siRNA, respectively. (**g**) Compared to siCon, the mRNA expression of Bax was obviously increased as Bcl-2 was obviously decreased in Cdc42, Nwasp or YAP knockdown podocytes. (**h–i**) Compared to siCon, the protein expression of Bax was obviously increased as Bcl-2 was obviously decreased in Cdc42, Nwasp or YAP knockdown podocytes. (**j**) A 24h-pooled urine sample was collected in a metabolic cage at day 0, 7, 14, 21 after the first injection of ML-141 for each mouse. Urinary albumin and creatinine were measured using a competitive ELISA. Proteinuria became obvious after injected with ML-141(Cdc42-specific inhibitor) for 14 days. *n*=5. (**k**) On top: typical images of immunochemistry-stained (red arrows point to WT-1-positive cells) glomeruli from control, Dmso and ML-141 mice. Below: representative micrographs of dual-color fluorescence staining of kidney glomeruli for WT-1 (red) and TUNEL (green) from control, Dmso and ML-141 mice. White arrows point to glomerular cells positive for both WT-1 and TUNEL (apoptotic podocyte), magnification × 400, scale bar=25 *μ*m. (**l**) Quantification of podocyte markers WT-1-positive cells per glomerular section. The number of WT-1-stained nuclei was obtained from 20 glomeruli per kidney section from five mice per group. (**m**) Absolute count of the numbers of glomerular cells positive for both TUNEL and WT-1 (white arrows), data were expressed as the means of apoptotic podocytes from 20 randomly selected glomeruli. All above data were from at least three independent experiments. *P<0.05 *versus* controls

To further explore the role of decreased Cdc42 in podocyte, we studied the proteinuria and podocyte apoptosis *in vitro* and *in vivo*. As shown in Figures 3a–f, apoptosis was markedly increased *in vitro*-cultured Cdc42 knockdown podocytes compare to siCon. We also investigated the mRNA and protein expression of Bax and Bcl2, which are two well-recognized indicators of apoptosis.^1^ As shown in Figures 3g–i, the mRNA and protein analysis of apoptosis signals Bax showed significantly increased expression in Cdc42-silenced podocytes, as apoptosis negative-related gene and protein Bcl-2 was inversely declined.

In addition, we surveyed podocytes in glomeruli by immunostaining with anti-WT-1 antibody, a podocyte-specific marker. As shown in Figures 3k and l, there was a marked reduction in glomerular WT-1-positive cells in glomeruli of mice treated with Cdc42-specific inhibitor ML-141, Furthermore, measurement of apoptosis cells by TUNEL (Figure 3k, below, 3M) staining showed that the number of apoptosis podocytes in glomerular was increased in mice treated with ML-141 comparing to the control mice (0.25±0.10 *versus* 2.05±0.11, *n*=3, *P*<0.05). Urinary albumin excretion was also significantly increased in ML-141-treated mice as compared with control mice (Figure 3j).

### Nwasp and YAP expression were decreased in injured podocytes

**Figure 4:**
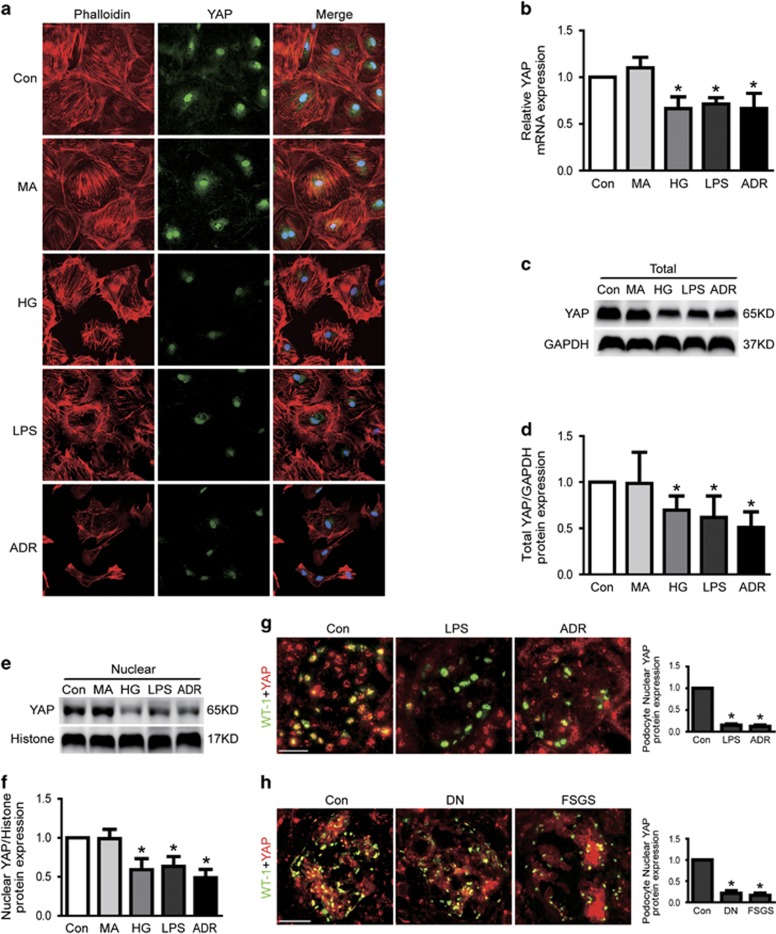
YAP and stress fiber were decreased in injured podocytes. (**a**) Typical confocal images of culture podocytes showing expression of YAP (green), Phalloidin-stained stress fiber (red) and DAPI-stained nuclei (blue). Compared to Con, nuclear and cytoplasmic YAP and stress fiber were obviously reduced in HG, LPS or ADR-injured podocytes. (**b**) The mRNA expression of YAP was decreased in HG, LPS or ADR-injured podocytes. (**c–f**) Total and nuclear protein expression of YAP was decreased in HG, LPS or ADR-injured podocytes. (**g**) Representative micrographs of dual-color fluorescence staining of kidney glomeruli for WT-1 (green) and YAP (red) from control, LPS and ADR mice. Magnification × 400, scale bar=25 *μ*m. (**h**) Representative micrographs of dual-color fluorescence staining of kidney glomeruli for WT-1 (green) and YAP (red) from control, DN and FSGS patients. Magnification × 400, scale bar=25 *μ*m. All above data were from at least three independent experiments. *P<0.05 *versus* controls

To further explore the underlying mechanism of Cdc42-induced podocyte apoptosis, we next investigated the expression of its downstream effector Nwasp and transcriptional co-activator YAP in HG or LPS or ADR-injured podocytes. As shown in Figures 2a–c, the results of real-time-qPCR and western blot analysis indicated that the mRNA and protein expression of Nwasp were markedly decreased in podocytes treated with HG or LPS or ADR. Results from immunofluorescence and western blotting demonstrated that both nuclear and cytoplasmic YAP protein expression was obviously decreased in injured podocytes *in vitro* and *in vivo* (Figures 4a, c–h). Similar results from PCR to analysis the mRNA expression of YAP were also obtained in HG or LPS or ADR-treated podocytes (Figure 4b). These data indicated that the Nwasp and YAP expression were significantly decreased in HG, LPS or ADR-injured podocytes.

### Loss of Nwasp or YAP-induced podocyte apoptosis *in vitro*

As lost of Cdc42 induced podocyte apoptosis, we investigated whether loss of its downstream effector Nwasp or YAP could induce podocyte apoptosis. As shown in Figures 3a–f, cell apoptosis rate was markedly increased *in vitro*-cultured Nwasp or YAP knockdown podocytes compared with siCon. Consistent with the results above, the mRNA and protein expression of Bax, a well-recognized indicator of apoptosis were significantly increased in Nwasp or YAP knockdown podocytes comparing to siCon (Figures 3g–i). However, Bcl-2, the indicator of anti-apoptosis was significantly decreased in Nwasp or YAP knockdown podocytes (Figures 3g–i).

### Overexpression of active YAP-alleviated podocyte apoptosis induced by HG, LPS or ADR

**Figure 5:**
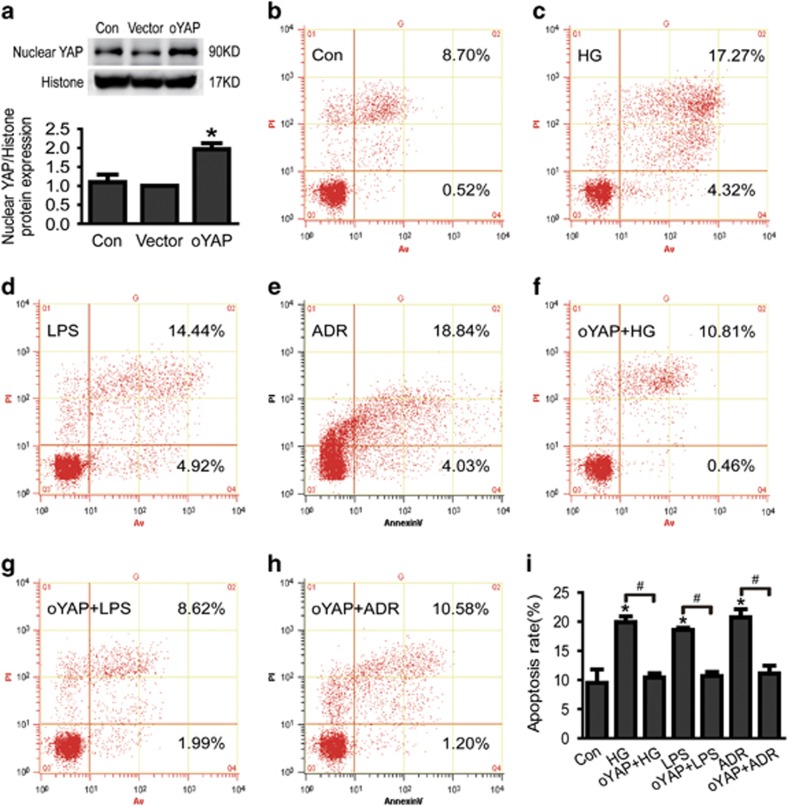
Overexpression of active YAP alleviates podocyte apoptosis induced by HG, LPS or ADR. (**a**) Nuclear YAP protein expression was obviously increased in overexpression of active YAP in podocytes. (**b–e**) Podocytes were stained with Annexin V/PI for flow cytometry analysis. Cell apoptosis rate was significantly increased in HG, LPS or ADR-treated podocytes. (**f–i**) Apoptosis induced by HG, LPS or ADR was significantly alleviated in overexpression of active YAP in podocytes. *P<0.05 *versus* Con, ^#^*P*<0.05

To further confirm the role of YAP in podocyte apoptosis, we investigated the effect of overexpression of active YAP in podocyte apoptosis induced by HG, LPS or ADR. Consistent with the results mentioned above, cell apoptosis rate was markedly increased *in vitro*-cultured podocytes treated with HG, LPS or ADR, respectively (Figures 5b–e). However, after overexpressed-active YAP *in vitro* podocytes, cell apoptosis induced by HG, LPS or ADR was significantly alleviated (Figures 5f–i). These results indicated that YAP mediated podocyte apoptosis was induced by HG, LPS or ADR.

### Loss of Cdc42/Nwasp-decreased YAP expression in podocytes

**Figure 6:**
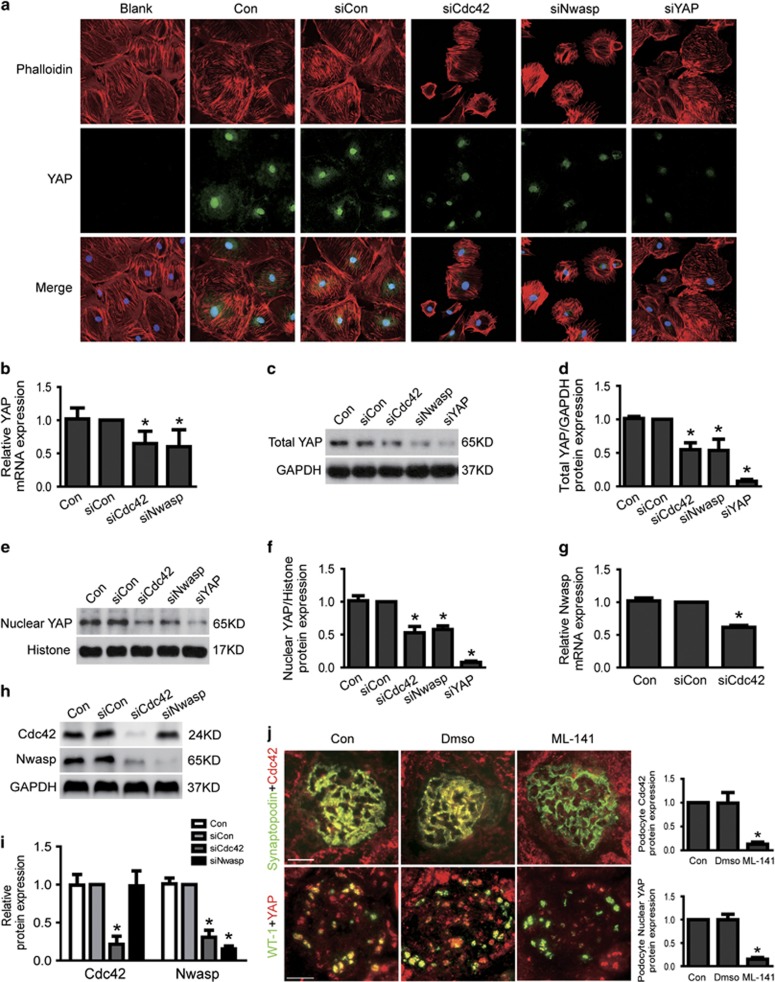
Loss of Cdc42/Nwasp decreased stress fiber formation and YAP expression. (**a**) Typical confocal images of culture podocytes showing expression of YAP (green), Phalloidin-stained stress fiber (red) and DAPI-stained nuclei (blue). Compared to siCon, nuclear and cytoplasmic YAP and stress fiber were obviously reduced in Cdc42 siRNA or Nwasp siRNA-treated podocytes. (**b**) The mRNA expression of YAP was decreased in Cdc42 siRNA and Nwasp siRNA-treated podocytes. (**c–f**) The total and nuclear protein expression of YAP was decreased in Cdc42 siRNA or Nwasp siRNA-treated podocytes. (**g**) The mRNA expression of Nwasp was decreased in Cdc42 siRNA-treated podocytes. (**h–i**) Cdc42 and Nwasp protein expression were reduced to about 21.8%, 15.4% in Cdc42 siRNA and Nwasp siRNA-treated podocytes, respectively. Nwasp protein expression was obviously decreased in Cdc42 knockdown podocytes comparing to siCon, as Cdc42 protein expression was not changed in Nwasp knockdown podocytes. (**j**) On top: typical images of dual-color fluorescence staining of kidney glomeruli for synaptopodin (green) and Cdc42 (red) from control, Dmso and ML-141 (Cdc42-specific inhibitor) mice. Cdc42 was hardly seen in podocytes from ML-141 mice. Below: representative micrographs of dual-color fluorescence staining of kidney glomeruli for WT-1 (green) and YAP (red) from control, Dmso and ML-141 mice. Nuclear YAP was decreased in podocytes from ML-141 mice comparing to Con. Magnification × 400, scale bar=25 *μ*m. All above data were from at least three independent experiments. *P<0.05 *versus* controls

To further explore the underlying mechanism of Cdc42-induced podocyte apoptosis, we also investigated the expression of its downstream effector Nwasp and YAP in Cdc42 knockdown podocytes. As shown in Figures 6g–i, the results of real-time-qPCR and western blot analysis indicated that the mRNA and protein of Nwasp were markedly decreased in Cdc42 knockdown podocytes. Results from both immunofluorescence and western blotting demonstrated that both nuclear and cytoplasmic YAP protein expression was obviously decreased in Cdc42 or Nwasp knockdown podocytes (Figures 6a, c–f). Similar results from real-time-qPCR to analysis the mRNA expression of YAP were also obtained in Cdc42 or Nwasp knockdown podocytes (Figure 6b). Furthermore, specifically inhibited Cdc42 expression with ML-141 in mice also decreased nuclear YAP protein expression (Figure 6j). Thus, these data strongly indicated that Cdc42/Nwasp could positively regulate YAP expression.

### Stress fiber/YAP-mediated Cdc42/Nwasp deficiency-induced podocyte apoptosis

To further explore the underlying mechanism of Cdc42/Nwasp regulating YAP, we firstly investigated the expression of Phalloidin-stained stress fiber, which had been regarded as a regulator of YAP,^20, 21^ in Cdc42 or Nwasp knockdown podocytes. As shown in Figure 6a, Phalloidin-stained stress fiber was decreased *in vitro* Cdc42 or Nwasp knockdown podocytes accompanied with increased podocyte apoptosis (Figures 3a–f). This was consistent with above results that stress fiber was also decreased (Figure 4a) as podocyte apoptosis was increased (Figures 1g–l) in HG, LPS or ADR-injured podocytes.

**Figure 7:**
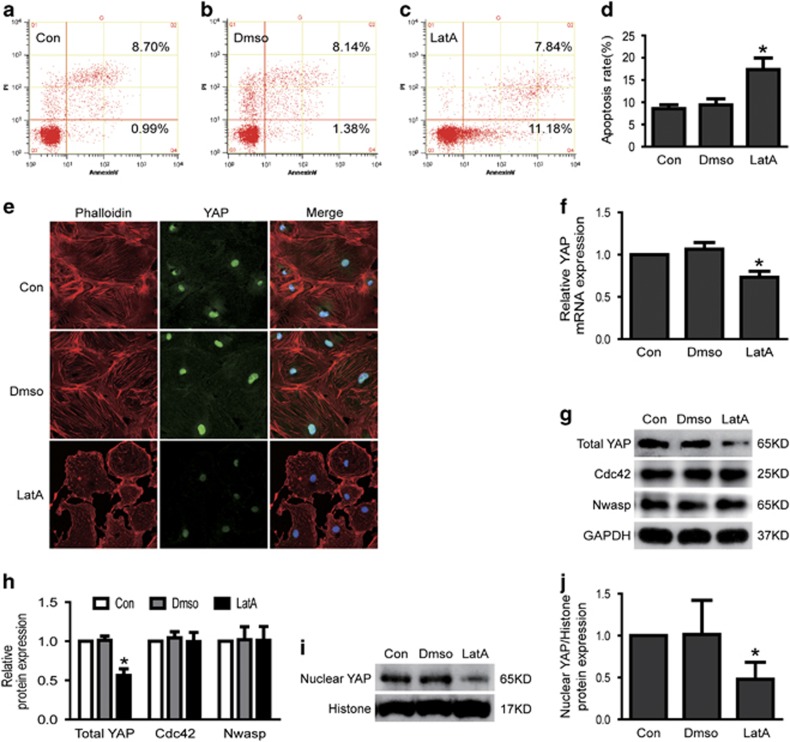
Inhibited formation of stress fiber induced podocyte apoptosis and decreased YAP expression. (**a–d**) Podocytes were stained with Annexin V/PI for flow cytometry analysis. Cell apoptosis rate was significantly increased in LatA-(F-actin inhibitor, inhibited stress fiber formation) treated podocytes. (**e**) Typical confocal images of culture podocytes showing expression of YAP (green), Phalloidin-stained stress fiber (red) and DAPI-stained nuclei (blue). Compared to Con, nuclear and cytoplasmic YAP and stress fiber were obviously reduced in LatA-treated podocytes. (**f**) The mRNA expression of YAP was decreased in LatA-treated podocytes. (**g–j**) Total and nuclear protein expression of YAP was decreased in LatA-treated podocytes, but the protein expression of Cdc42 and Nwasp were not changed. All above data were from at least three independent experiments. *P<0.05 *versus* controls

**Figure 8:**
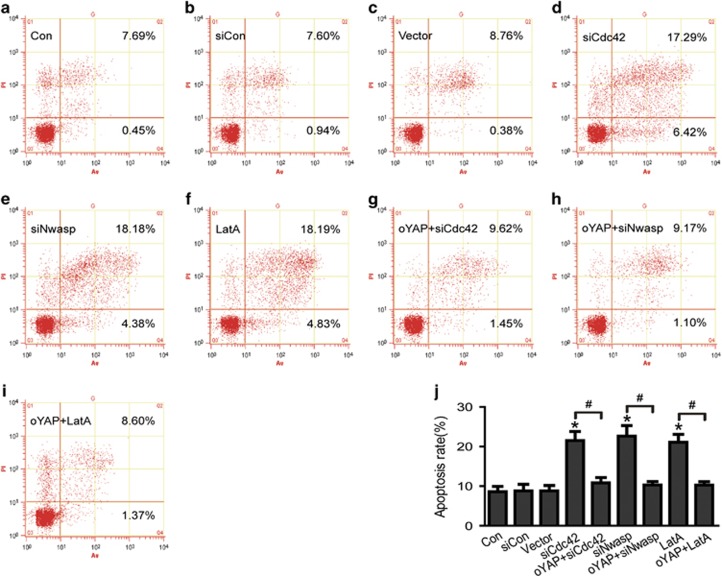
Overexpression of active YAP decreased podocyte apoptosis induced by Cdc42 or Nwasp knockdown podocytes or LatA-treated podocytes. Podocytes were stained with Annexin V/PI for flow cytometry analysis. Con, siCon, vector, oYAP, siCdc42, siNwasp, siYAP were short for blank control, control siRNA, overexpression vector, overexpressed-active YAP, Cdc42 siRNA, Nwasp siRNA, YAP siRNA, respectively. (**a–f**) Cell apoptosis rate was significantly increased in Cdc42 or Nwasp knockdown podocytes or LatA-(F-actin inhibitor, inhibited stress fiber formation) treated podocytes. (**g–j**) Podocyte apoptosis induced by Cdc42 or Nwasp knockdown or LatA treatment was significantly inhibited by overexpressing active YAP. All above data were from at least three independent experiments. *P<0.05 *versus* controls, ^#^*P*<0.05

Then, we explored the role of stress fiber in podocytes apoptosis. As shown in Figures 7a–j, inhibited stress fiber formation with the specific inhibitor LatA resulted in increased podocyte apoptosis accompanied with decreased YAP mRNA and protein expression, but not changed the protein expression of Cdc42 and Nwasp. To further confirmed Cdc42/Nwasp/stress fiber-mediated podocyte apoptosis through YAP, we also investigated the apoptotic effect of overexpression of active YAP in Cdc42 or Nwasp knockdown podocytes or LatA-treated podocytes. As shown in Figures 8a–j, podocyte apoptosis induced by Cdc42 or Nwasp knockdown or LatA treatment was significantly abolished by overexpressing active YAP. These data mentioned above indicated that the stress fiber/YAP probably mediated Cdc42/Nwasp deficiency, induced podocyte apoptosis.

## Discussion

Podocytes are very special cells with complex actin cytoskeletal architecture, which play a pivotal role in maintaining normal glomerular function.^25^ Loss of podocyte caused by apoptosis is commonly happened in the pathogenesis of many kidney diseases, such as diabetic nephropathy,^2, 3^ focal segmental glomerular sclerosis,^4, 26^ *et al.* However, the mechanism-mediating podocyte apoptosis remains incompletely understood. Therefore, exploring the mechanisms that cause podocyte apoptosis in these kidney diseases is imperative.

In this study, we found that Cdc42 expression and its activity were significantly decreased in injured podocytes induced by HG or LPS or ADR. Decreased Cdc42 expression is also observed in podocytes of patients with diabetic DN or FSGS. Moreover, we found that podocyte apoptosis was increased in Cdc42 deficiency podocytes *in vivo* and *in vitro*. We also further demonstrated that the loss of Cdc42 or its downstream effector Nwasp-induced podocyte apoptosis was probably mediated by decreased stress fibers/YAP expression, which the latter had been regarded as an anti-apoptosis protein in podocyte.^18, 19^ Collectively, these data indicated that the Cdc42/Nwasp/stress fibers/YAP signal pathway may potentially play an important role in regulating podocyte apoptosis.

The function of podocytes is regulated by small GTPases belonging to the Rho GTPase family.^7^ These small GTPases act as molecular switches to control the activation of multiple downstream effectors.^27, 28, 29, 30^ Among their pleiotropic actions, Rho-dependent signaling cascades modulate cellular morphology and actin polymerization, adhesion, cell migration, proliferation and apoptosis.^20, 27, 28, 29, 30, 31^ Cdc42 is one of the Rho-GTPases that have been well studied, and has been regarded as a main regulator of the function of cytoskeletal architecture.^11, 12^ It mediates multiple signaling pathways, including tyrosine kinase receptors, heterotrimeric G-protein-coupled receptors, cytokine receptors, integrins, and physical and chemical stress.^32^ Recent studies demonstrated Cdc42 has a crucial role in podocyte cell maintenance.^33^ Mice only lacking Cdc42 showed early embryonic lethality and defective actin cytoskeleton.^11^ Podocyte-specific deletion of Cdc42, but not Rac1, resulted in many overt changes to podocyte morphology and overall glomerular function.^12^ Here, in our study, we firstly focused on the response of Cdc42 to podocyte injury. We investigated the expression of Cdc42 in human nephropathy with proteinuria. Interestingly, Cdc42 protein expression and activity were significantly decreased or inhibited in injured podocyte induced by HG, LPS and ADR. Similarly, decreased Cdc42 expression was also detected in glomerular podocyte of FSGS and DN patients.

Based on pleiotropic actions of Cdc42,^20, 31^ we speculated that the reduction of Cdc42 in the podocyte as a response to injury might be related to podocyte apoptosis. Therefore, we next sought to investigate whether the loss of Cdc42 induces podocyte apoptosis. Indeed, we observed increased apoptotic podocytes by both TUNEL assay and Annexin V, and propidium iodide staining after knockdown of endogenic Cdc42 gene *in vitro* and inhibition of Cdc42 activity *in vivo*. Meanwhile, the number of podocytes was significantly decreased after inhibition of Cdc42 activity with ML-141 *in vivo*. In addition, apoptosis-related genes Bax and Bcl2 were also assessed by real-time PCR and immunoblot, and significant aberrant expressions were found. These data demonstrated that insufficient Cdc42 could cause podocyte apoptosis, which is another main reason that will cause proteinuria. This indicates that the podocyte apoptosis induced by Cdc42 deficiency is probably a new mechanism of proteinuria.

To further explore the mechanisms of Cdc42 deficiency-mediated podocyte apoptosis, we investigated the expression of its downstream effector Nwasp and transcriptional co-activator YAP.^34^ Numerous studies have demonstrated that the Rho GTPase Cdc42 controls actin dynamics by regulating actin polymerization stability via its downstream target Nwasp *in vitro*.^30, 35^ In present study, we found that Nwasp protein expression was significantly decreased in Cdc42 knockdown podocytes, however, Cdc42 protein expression was not changed in Nwasp knockdown podocytes, it was consistent with previous results that the Nwasp protein was downstream target of Rho GTPase Cdc42. In addition, we also demonstrated that apoptosis was increased in Nwasp knockdown podocytes. The apoptosis-related marker Bax and Bcl2 were also found to be activated and inhibited respectively accompanied with decreased Cdc42 and Nwasp. These further suggest that Cdc42/Nwasp is implicated in podocytes apoptosis.

YAP is a transcriptional co-activator that regulates cell proliferation and apoptosis downstream of the Hippo kinase pathway. Dephosphorylated YAP localizes in the nucleus and functions as a transcriptional co-activator to induce target gene expression in normal condition.^15^ It has a well-described role in controlling the expression of genes that promote cell proliferation and inhibit apoptosis.^13, 14, 18^ In the current study, we found that the total and nuclear YAP protein expression were significantly decreased in HG, or LPS or ADR-mediated podocyte injury *in vivo* and *in vitro*. Similar results were also obtained in patients with DN and FSGS. Evidence had showed that YAP could also serve as a physiologic inhibitor of podocyte apoptosis.^18^ This was consistent with our results that apoptosis was increased in YAP knockdown podocytes. In addition, the apoptosis-related marker Bax and Bcl2 were also found to be aberrant in YAP knockdown podocytes.

Previous studies had identified YAP/TAZ as sensors and mediators of mechanical cues instructed by the cellular microenvironment.^21, 36^ Cdc42/Nwasp is also known to regulate the dynamic organization of the cytoskeleton.^30, 34^ Therefore, it's possible that Cdc42/Nwasp could positively regulate YAP in podocytes. As anticipated, our data further demonstrated that both nuclear and cytoplasmic protein expression of YAP were decreased in Cdc42 or Nwasp knockdown podocytes. Similar results were also obtained in glomerular podocyte of mice treated with specific Cdc42 inhibitor ML-141.^37^ It is consistent with the previous studies that Rho-GTPases could positively regulate YAP transcriptional activity^21^ and protein expression.^38^ Although previous study also showed that the inhibition of Cdc42 caused YAP inhibition, mainly by YAP sequestration in the cytoplasm in mouse embryonic fibroblasts,^20^ it's possible that Cdc42 could positively regulate YAP expression in podocytes. However, the mechanism remains to study further. Whatever, these data further suggests that Cdc42/Nwasp/YAP is probably implicated in podocyte apoptosis.

To further explore the underlying mechanism of Cdc42/Nwasp regulate YAP, we focus on a cytoskeleton molecule Phalloidin-stained stress fiber, which had been regarded as a regulator of YAP.^21^ Previous studies had showed that Cdc42 could work through actin cytoskeleton to promote stress fibers formation. Similarly, in our study we found that podocyte deficiency in Cdc42 or Nwasp could significantly lead to loss of stress fiber, decreased YAP expression and increased podocyte apoptosis. This was consistent with results that stress fiber was also decreased in HG, LPS or ADR-treated podocytes with increased podocyte apoptosis. Inhibited stress fiber with specific inhibitor LatA^21^ resulted in increased podocyte apoptosis accompanied with decreased nuclear and cytoplasmic YAP expression, but not changed the protein expression of Cdc42 and Nwasp. These results indicated that YAP was the downstream molecule of stress fiber, but not Cdc42 and Nwasp.

To further confirmed Cdc42/Nwasp/stress fiber-mediated podocyte apoptosis through YAP, we also investigated the apoptotic effect of overexpression of active YAP in Cdc42/Nwasp/stress fiber deficiency podocytes. We found that the podocyte apoptosis induced by Cdc42 or Nwasp knockdown or LatA treatment was significantly alleviated by overexpressing active YAP. These results indicated that stress fiber/YAP probably mediated Cdc42/Nwasp deficiency-induced podocyte apoptosis. Therefore, Cdc42/Nwasp/stress fibers/YAP may be an important signal pathway to regulate podocyte apoptosis. Insufficient Cdc42/Nwasp/stress fibers/YAP pathway activity in podocyte may be one common reason that causes proteinuria in kidney diseases including FSGS and DN.

Taken together, the present study demonstrated that the expression of Cdc42 and its activity were significantly decreased as cell apoptosis was increased in injured podocyte *in vitro* and *in vivo*. Decreased Cdc42 expression by siRNA and selective inhibitor ML-141 *in vitro* and *in vivo* caused podocyte apoptosis and proteinuria, accompanied with increased pro-apoptotic Bax and decreased anti-apoptotic Bcl-2 gene and protein expression. Our results further demonstrated that insufficient Cdc42 or Nwasp, its downstream effector, could decrease the mRNA and protein expression of YAP, which had been regarded as an anti-apoptosis protein in podocyte. Moreover, our data indicated that loss of stress fibers caused by Cdc42/Nwasp deficiency also decreased YAP mRNA and protein expression, and induced podocyte apoptosis. Podocyte apoptosis induced by Cdc42/Nwasp/stress fibers deficiency was significantly abolished by overexpressed-active YAP. Therefore, the Cdc42/Nwasp/stress fibers/YAP signal pathway may potentially play an important role in regulating podocyte apoptosis. Maintaining necessary Cdc42 would be one potent way to prevent proteinuria kidney diseases.

## Materials and Methods

### Cell culture and treatment

The conditionally immortalized mouse podocyte cell line was a kind gift from Dr Jochen Reiser (Rush University Medical Center, Chicago, IL, USA), and cultured as described previously.^39^ Cells were cultured at 33 °C in RPMI-1640 medium (Gibco BRL, Gaithersburg, MD, USA) supplemented with 10% fetal bovine serum (FBS, Gibco BRL, Gaithersburg, MD, USA) and recombinant IFN-*γ* (growth permissive conditions; CYT-358, ProSpec, Tany Technogene Ltd, Ness Ziona, Israel). To induce differentiation, podocytes were reseeded and cultured at 37 °C in 100 cm^2^ culture dish coated with 12 mg/ml type-I collagen (BD Bioscience, Bedford, MA, USA) and in Ddulbecco's modified eagle medium (with 5.3 mM glucose, Invitrogen, Carlsbad, CA, USA) supplemented with 5% FBS, deprived of IFN-*γ* (growth restrictive conditions) for 10–13 days. After differentiation, podocytes was confirmed by the identification of synaptopodin, a podocyte differentiation marker, 10^6^ cells were synchronized into quiescence by growing cells in serum-free RPMI-1640 medium (Gibco BRL) for 24 h, and then treated with MA, HG (add to 30 mM glucose), LPS (100 *μ*g/ml), ADR (0.125 *μ*g/ml) or siRNA (50 nM) for 48 h. Each reaction was repeated in triplicates.

### Transfection of siRNAs and plasmids

The siRNAs against Cdc42, Nwasp, YAP, and control were designed and synthesized by RiboBio Co. Ltd (Guangzhou, China). They were transfect into podocytes using Lipofectamine 2000 reagent (Invitrogen) following the manufacturer's protocol. The sequences of siRNAs used in this study were as follows: siCdc42 5′GCAAGAGGAUUAUGACAGA dTdT-3′, siNwasp 5′GGUGUCGCUUGUCUGGUUA dTdT-3′, siYAP 5′-CGAGAUGAGAGCACAGACA dTdT-3′. Lentiviral packaging wild-type YAP or YAP S112A mutation plasmids (CS-Mm06093-Lv128, GeneCopoeia, Guangzhou, China) were generated in HEK293T cells that were expanded and transfected using the Lenti-Pac HIV Expression Packing Kit (HPK-LvTR-20, GeneCopoeia) according to the manufacturer's protocols. Podocytes were then infected in 6-well plate (Trueline, Salt Lake, UT, USA) with the lentivirus particles. Forty eight hours after transfection, the cells under different conditions were collected. Each transfection was repeated in triplicate.

### Real-time quantitative-PCR

As described previously,^2^ RNA samples were prepared using Trizol RNA isolation system (Invitrogen) and reverse transcribed into cDNAs using the PrimeScriptTM RT regent Kit following the instructions provided by the manufacturer (Takara Bio Inc, Otsu, Shiga, Japan), and then cDNAs were used for real-time PCR analysis by a Plantinum SYBR Green SuperMix-UDG kit (Takara Bio Inc). The primers used are listed as follows: Cdc42, forward 5′-ATTATGACAGACTACGACCGCT-3′, reverse 5′-AGTGGTGAGTTATCTCAGGCA-3′ Nwasp, forward 5′-ATGCTCCAAATGGTCCCAATC-3′, reverse 5′-CTTGGTGTTCCAATATCTGCCT-3′ YAP, forward 5′-TGAGATCCCTGATGATGTACCAC-3′, reverse 5′-TGTTGTTGTCTGATCGTTGTGAT-3′ Bax, forward 5′-CTGGACCATAGGTCGGAGTG-3′, reverse 5′-AATTCGCCGGAGACACTCG-3′ Bcl-2, forward 5′-GTCGCTACCGTCGTGACTTC-3′, reverse 5′-CAGACATGCACCTACCCAGC-3′ GAPDH, forward 5′-AGGTCGGTGTGAACGG ATTTG-3′, reverse 5′-TGTAGACCATGTAGTTGAGGTCA-3′.

### Western blotting

Whole and nuclear proteins were prepared as described previously.^2^ An aliquot of cell lysates containing 30 *μ*g of protein was separated on 10% sodium dodecyl sulfate–polyacrylamide gels, and then transferred to polyvinylidene fluoride membranes (Millipore, Billerica, MA, USA). After blocked by 5% non-fat dry milk for 1 h at room temperature, membranes were incubated overnight at 4 °C with the following primary antibodies: rabbit anti-YAP (Cell Signaling Technology, Danvers, MA, USA, 1:1000), rabbit anti-Histone (Cell Signaling Technology, 1:3000), rabbit anti-Cdc42 (Santa Cruz, Dallas, TX, USA, 1:500), rabbit anti-Nwasp (Santa Cruz, 1:500), rabbit anti-Bax (Santa Cruz, 1:500), rabbit anti-Bcl-2 (Cell Signaling Technology, 1:1000), rabbit anti-GAPDH (Bioworld Technology, Nanjing, China, 1:10 000). The membranes were then incubated with anti-rabbit IgG (Jackson Immuno Research, West Grove, PA, USA, 1: 4000) at room temperature for 1h. Finally, membranes were treated with ECL reagents (Advansta, Menio Park, CA, USA), followed by exposing to X-ray film (Kodak, Rochester, NY, USA). The bands of the resulting autoradiographs were quantified densitometrically using Bandscan software. Protein expression was quantified as the ratio of specific band to Histone (nuclear fractions) or GAPDH.

### Annexin V and propidium iodide (PI) staining assay

Apoptotic cells in different groups were determined using an Annexin V/PI apoptosis detection kit according to manufacturer's protocol (Nanjing KeyGEN Biotech, Nanjin, China). Briefly, podocytes were resuspended with binding buffer followed by incubation with 5 ml of Annexin V (conjugated with FITC) and 5 ml of PI in the dark for 10 min. Cell fluorescence was then analyzed using a Cell Lab QuantaTM SC Flow cytometer (Beckman Colter, Inc, USA). Cells positive for Annexin V-FITC were considered as apoptosis.

### Cdc42 GTPase activation assay

Active Cdc42 was measured by G-LISA Cdc42 Activation Assay Biochem kit (colorimetric assay, Cytoskeleton, Denver, CO, USA) following the manufacturer's instruction. And the signal was measured at 490 nm with a microplate reader (MRX, Dynatech Laboratories, Chantilly, VA, USA). Total Cdc42 protein expression of each group was measured by WB, as described above. Cell lysate from each group containing the same amount of protein was measured in both Cdc42 GTPase activation assay and WB. Results were expressed as fold activity of stimulated in relation to non-stimulated controls normalized to total Cdc42 protein content.

### Patient studies

Renal biopsies tissues from patients with DN, FSGS and renal cell carcinoma (adjacent normal tissues) were respectively obtained after patients gave written consent. The study of patients was conducted in accordance with the Second Helsinki Declaration and approved by the Ethics Committee for Human Research of Guangdong General Hospital.

### Animal studies

Animal care and experiments were performed in accordance with the ARRIVE guidelines,^40^ and were approved by the Ethics Committee for animal research of Guangdong General Hospital. Six male C57BL/KsJ db/db mice and 5 age-matched wild-type (BKS) mice were purchased from Model Animal Research Center of Nanjing University. Thirty-six male BALB/c mice aged 8 weeks were purchased from Center of Laboratory Animal Science of Guangdong. Mice were anaesthetized (ketamine, 70 mg/kg, intraperitoneally (i.p.)) before killed and kidney tissue were collected. Mice urinary albumin and creatinine were measured using mouse albumin-specific ELISA (Bethyl Laboratories Inc, Montgomery, TX, USA) and creatinine kits (Cayman Chemical, Ann Arbor, MI, USA) respectively, according to the manufacturer's instructions. Proteinuria was expressed as the ratio of albumin to creatinine.

### Diabetic mice model

The C57BL/KsJ db/db mice were obese and known to develop type 2 diabetes, followed by diabetic kidney disease. For urine collection, individual mice were caged once every 2 weeks in a metabolic cage for 24 h from week 12–week 20.

### Murine models of LPS-induced proteinuria and podocytopathy

Five male BALB/c mice were randomized to each of the following treatments: LPS-induced proteinuric mice were i.p. injected with 200 *μ*g LPS (1 mg/ml in sterile LPS-free saline, serotype: *E*. *coli* 0111:B4, Sigma, St Louis, MO, USA) in a total volume of 200 *μ*l; control mice were i.p. injected with equal volumes of sterile LPS-free saline.^41^ Mice were followed for 24 h before they were killed and the kidneys resected for further investigation. Urine was collected before and 24 h after LPS injection.

### Murine models of ADR-induced proteinuria and podocytopathy

Five male BALB/c mice were injected once with ADR (2 mg/ml in sterile ADR-free saline, doxorubicin hydrochloride, Sigma) at a dose of 12 mg/kg body weight via the tail vein on day 0. Five control BALB/c mice were injected with an equal volume of saline only.^42^ Urine of all mice was collected on day 0, 4, 7, 14.

### Murine models of Cdc42 inhibition

Five male BALB/c mice were i.p. injected with 100 *μ*l ML-141 (selective inhibitor of Cdc42, 1 mg/ml in 30% dimethylsulfoxide-saline, Tocris Bioscience, Bristol, UK) daily for 3 weeks.^37, 43, 44^ Five blank control mice and five DMSO control mice were i.p. injected with 100 *μ*l saline and 30% dimethylsulfoxide-saline respectively everyday for 3 weeks. Urine of all mice was collected weekly before killed.

### Podocyte number counting

The number of podocytes per glomerulus was determined based on methods described previously.^45, 46^ Paraffin embedded tissue sections were deparaffinized and hydrated with water, then were placed in preheated 10 mM Tris-buffered saline with 1.0 mM EDTA (pH 9.0) and heated for 20 min at 100 °C in a water bath. After cooling to room temperature (RT), slides were rinsed in deionized water and then immersed in 3% H_2_O_2_ to quench endogenous peroxidase activity. After being washed, slides were incubated with rabbit polyclonal antibody Wilm's tumor antigen 1 (WT-1; sc-192, Santa Cruz) for 60 min at RT, then washed and immunoperoxidase staining was performed using the Envision Plus system for rabbit primary antibodies (Dako Cytomation, Carpinteria, CA, USA) according to the manufacturer's instructions. The slides were counterstained with haematoxylin and permanently mounted before examination by light microscopy. The number of podocytes per glomerulus is the average number of nuclei stained in 20 randomly selected glomeruli.

### Immunofluorescent staining and TUNEL staining

Cultured podocytes planted on cover slides in six-well plates or frozen cryostat sections were fixed with 4% paraformaldehyde at RT for 20 min, permeabilized with 0.1% Triton X-100 for 5 min, then blocked with 5% bovine serum albumin for 30 min at room temperature before further incubated overnight at 4 °C with the following primary antibodies: goat anti-synaptopodin (Santa Cruz, 1:100), goat anti-WT-1 (Santa Cruz, 1:100), rabbit anti-Cdc42 (Santa Cruz, 1:100), rabbit anti-YAP (Santa Cruz, 1:100). Then cultured podocytes or sections were washed three times with PBS for 5 min before the secondary antibodies (FITC-donkey anti-goat IgG 488, Protein Tech Group, Inc, Rosemont, IL, USA, 1:200; goat anti-rabbit Alexa Fluor 555, Cell Signaling Technology, 1:200; goat anti-rabbit Alexa Fluor 488, Cell Signaling Technology, 1:1000) were applied for 1 h at RT in the dark. Culture podocytes were stained with phalloidin (Cytoskeleton, 1:500) for 30 min and then with DAPI (Sigma, St. Louis, MO, USA) for 5 min at room temperature. Apoptotic cell death in kidney sections was detected by using the TUNEL kit (Roche Molecular Biochemicals, Mannheim, Germany) as described previously.^41, 47^ Cells positive for both TUNEL and WT-1 are apoptotic podocytes. The quantifications of these data were expressed as the means of apoptotic podocytes from 20 randomly selected glomeruli. Photomicrographs were taken with laser confocal microscopy (LCSM, Zeiss KS 400, Postfach, Germany) and analyzed by Image-Pro Plus 6.0 (Media Cybernetics, Georgia Avenue, MD, USA) for quantification. All images were analyzed by two investigators blinded to the identity of the samples.

### Statistical analysis

All values are expressed as mean±S.D. Statistical analysis was performed using the statistical package SPSS for Windows Ver. 19.0 (SPSS, Inc., Chicago, IL, USA). All experimental observations were repeated more than three times. Statistical analysis of the data from multiple groups was performed by analysis of variance followed by Student–Newman–Keuls tests. Data from two groups were compared by Student's *t*-test. *P*-values <0.05 were considered significant.

## Abbreviations

ADR: adriamycin
HG: high glucose
LPS: lipopolysaccharide
Nwasp: neuronal Wiskott–Aldrich syndrome protein
siRNA: small interfering RNA
YAP: Yes-associated protein
